# Is gender policy related to the gender gap in external cause and circulatory disease mortality? A mixed effects model of 22 OECD countries 1973–2008

**DOI:** 10.1186/1471-2458-12-969

**Published:** 2012-11-12

**Authors:** Mona Backhans, Bo Burström, Antonio Ponce de Leon, Staffan Marklund

**Affiliations:** 1 Department of Public Health Sciences, Karolinska Institutet, Stockholm, 171 76, SWEDEN; 2 Department of Epidemiology, Instituto de Medicina Social, Rio de Janeiro State University, Rio De Janeiro, BR-20550011, Brazil; 3 Division of Insurance Medicine, Department of Clinical Neuroscience, Karolinska Institutet, Stockholm, 171 76, SWEDEN

**Keywords:** Gender policy, Gender equality, Mortality gaps, Country comparison, Time series data

## Abstract

**Background:**

Gender differences in mortality vary widely between countries and over time, but few studies have examined predictors of these variations, apart from smoking. The aim of this study is to investigate the link between gender policy and the gender gap in cause-specific mortality, adjusted for economic factors and health behaviours.

**Methods:**

22 OECD countries were followed 1973–2008 and the outcomes were gender gaps in external cause and circulatory disease mortality. A previously found country cluster solution was used, which includes indicators on taxes, parental leave, pensions, social insurances and social services in kind. Male breadwinner countries were made reference group and compared to earner-carer, compensatory breadwinner, and universal citizen countries. Specific policies were also analysed. Mixed effect models were used, where years were the level 1-units, and countries were the level 2-units.

**Results:**

Both the earner-carer cluster (ns after adjustment for GDP) and policies characteristic of that cluster are associated with smaller gender differences in external causes, particularly due to an association with increased female mortality. Cluster differences in the gender gap in circulatory disease mortality are the result of a larger relative decrease of male mortality in the compensatory breadwinner cluster and the earner-carer cluster. Policies characteristic of those clusters were however generally related to increased mortality.

**Conclusion:**

Results for external cause mortality are in concordance with the hypothesis that women become more exposed to risks of accident and violence when they are economically more active. For circulatory disease mortality, results differ depending on approach – cluster or indicator. Whether cluster differences not explained by specific policies reflect other welfare policies or unrelated societal trends is an open question. Recommendations for further studies are made.

## Background

This study relates to three strands of research: studies that associate social policy to population health and health inequalities, studies on the impact of gender equality on population health, and studies that investigate trends and country differences in the male/female mortality gap.

### Comparative policy studies with a health focus

As welfare policies have clear effects on income distribution and poverty outcomes [1-3], it would seem likely that they also have effects on the health of the population, as well as health disparities. Some researchers have investigated differences between country clusters, either defined by their political tradition [4,5] or their welfare regime type [6-8]^a^. Studies on either cluster or country differences tend to find few significant differences favouring social democratic welfare regimes in terms of health inequalities defined according to education, social class or income [9-13] although there are exceptions [5,8]. Instead, often the corporatist countries have the smallest (relative) differences. Some studies have however found cluster differences in absolute levels of health favouring the Nordic countries [6,7,14] and there are indications that the Nordic welfare state may buffer against detrimental effects of economic recession [10]. Instead of clustering countries, Navarro has included accumulated years of political incumbency as a predictor, and found that reduced infant mortality is clearly related to years of government of redistributive parties [15]. Also Muntaner [16] found consistent associations between indicators of working class strength and measures of birth and infant survival.

A different analytic strategy is to investigate specific policies and their impact on population health. Factors identified as strongly associated with health improvements are total social spending, universal access to social insurance [17], parental leave and the generosity of basic security pensions [18]. One study found that social welfare spending led to a reduction of cause-specific mortality, whereas healthcare spending did not [19] but other researchers have found medical coverage and primary care to be important for health outcomes [16,20,21]. The idea behind this strategy, as argued by Lundberg [22], is that it is the existence and level of certain welfare state institutions that explain cluster differences and that crude categorisations fail to identify the specific welfare state characteristics that matter for health. He also criticises studies that investigate political party incumbency rather than what parties do. This critique receives some support from Chung & Muntaner [20], who found that the percentage of left vote lost its explanatory power when welfare state variables were entered in the model. Others have argued that the effect of politics goes beyond policy e.g. through social processes such as grass root movements and NGOs, and that studying policies one by one conceals possible inter-sectoral effects [23]. A recent review of 73 comparative studies has shown that the factors most consistently related to good health was the strength of democracy and egalitarian political traditions, whereas studies using a welfare regime framework more often found mixed results [24].

Research on the health effects of gender policy, or the effect of policy on gender differences in health, is rare. A group of researchers, however, have examined the association between US state-level policies and women’s health. They found that access to health insurance and services, gun control, and policies on violence against women were related to female mental health and cause-specific mortality [25], and that access to health care, policies on violence against women and antidiscrimination policies were associated with blood pressure, smoking and obesity [26]. Bambra et al. [27] have examined the relationship between gender and self-assessed health in 13 countries categorised according to an expanded welfare state framework [28]. The study showed that while women in the social democratic and Southern welfare states were more likely to report worse health than men, there were no gender differences in the corporatist countries. Possible causes of the poor performance of the social democratic countries were, according to the authors, women’s dual roles in countries with high female labour force participation, combined with a sex segregated labour market offering worse jobs for women.

### Gender equality as a health determinant

Aggregate and multilevel studies from the USA have examined gender equality on state level as a determinant of health. These have used indices of women’s political participation, economic autonomy, employment and earnings, and reproductive rights as indicators of gender equality. Investigated outcomes were mortality and reported days of activity limitations [29], women’s self-rated health [30], depressive symptoms [31], and child well-being [32]. These studies have found that states that perform poorly on the gender equality indicators also have worse health outcomes, for men, women and children. A previous Swedish study, however, found negative associations between gender equality (measured as political participation, division of labour and economic resources) at municipal level and health for both men and women, while results regarding gender inequalities in health were inconclusive [33]. These discordant results (cp to previous studies) were primarily attributed to an unbalanced mix of high gender equality regarding political participation and income, but with large remaining inequalities in the division of labour, both paid (i.e. sex-segregation) and unpaid. An international study of adolescent’s health showed that health complaints in both boys and girls were lower in countries with a high Gender Empowerment Measure (GEM)^b^, and that the gender gap in complaints was larger in countries with a low Gender-related Development Index^c^[34]. Another study focusing on male mortality used a sample of 51 countries from four continents and found a strong association between female homicide rates, seen as an extreme expression of patriarchy, and mortality [35]. Thus, gender equality overall is positively associated with health and may also contribute to smaller gender gaps in health. However, as the studies by Bambra et al. [27] and Backhans et al. [33] have shown, the relationship may sometimes be reversed.

### The male/female mortality gap

In countries where very long time-series data is available it has been shown that from the early 17th to the early 20th century male and female mortality differed only slightly, with absolute differences varying from 0 to 2 years of life expectancy at birth [36,37]. In some age groups, there was a male advantage, due to a gender unequal resource allocation and high maternal mortality [38]. During the 20^th^ century, life expectancy for both men and women has been steadily rising [39]. From 1950 the male/female mortality gap has increased and then decreased, with the peak year varying from the early 1970s to the 1990s and with some countries still seeing no decline [40]. The largest decline is found among the middle-aged (55–75 years) and the causes of death that have contributed most to the decline in the mortality gap are heart disease, accidents and violence (excluding suicide), lung cancer and breast cancer [41].

There is a scientific controversy regarding the causes of the changing gender gap in mortality. Some scholars argue that changes are primarily driven by the stage of diffusion of cigarette smoking [42], unrelated to changes in women’s roles and relative status [43-46]. Others point out the high association between the GEM and the male/female smoking ratio [47], and it has been suggested that female emancipation is the underlying factor behind widespread take-up of smoking [48]. Smoking has been found to be more common among highly educated women 60 years and older, but with a reverse pattern in women 25–39 years [49], reflecting the take-up of and abandonment of smoking through hierarchical diffusion, where the lifestyles of dominant groups are gradually adopted by the whole population, while the former continue to change and refine their consumption style [50,51] One factor halting diffusion could be a high degree of gender inequality, making it less likely that behaviours are seen as either affordable or appropriate [52]. Groups remote in social space are unlikely to influence each other’s habits directly [53]. Therefore, female emancipation can be seen as a prerequisite for the adoption of (formerly) masculine behavioural patterns.

Some researchers, especially those with a biological/evolutionary outlook, have chosen to focus on risk-taking among (young) males as a primary explanation of gender differences in mortality [37,54]. Data regarding the mortality gap in external causes shows that since ca 1940 there has been a faster mortality decline among women than men, followed by greater improvements among young men [37,41]. Waldron [55] examined trends in gender differences in accident mortality in five large OECD countries 1950–1998 and concluded that a combination of convergence of gender roles and the differential impact (due to existing gender differences) of other societal trends (e.g. regarding drug use and improvements in medical care or public health measures) could account for most trends.

To summarise, few studies have examined the impact of policy on gender differences in health, or of gender policy on the absolute levels of health or disease. Bambra et al. [27] utilised a standard (not gender-focused) welfare policy framework, and this may arguably not be as relevant when gender gaps rather than social inequalities are studied. As far as we are aware, no previous study has examined the association between gender policy and the gender mortality gap, thus linking these two research strands together.

### Aims and hypotheses

The aim of this study is to investigate the link between gender policy and the gender gap in external cause and circulatory disease mortality. These outcomes were chosen as they both contribute strongly to the overall gender gap in mortality and to its decline, while their association with smoking is different.

Our main hypothesis is that earner-carer countries [56] should have smaller and/or decreasing gender gaps in mortality, and that this difference is primarily due to the particular policies that distinguish them - policies which are employment-supporting for women/mothers, lessen the caring burden of families, support fathering, and decrease the effect of previous employment on economic conditions in old age. The association should to a large part be mediated through achieved gender equality, e.g. through convergence of women’s and men’s status, gender roles and health behaviours [33].

In the case of external cause mortality, women’s role expansion is likely to lead to increased risk exposure, and probably also to more risk-prone behaviour. For men, gender equality-friendly countries may be characterised by masculinities that are less ‘extreme’ and thus less risk-prone, than in more traditional countries [57].

In the case of circulatory disease mortality, women’s role expansion could be both health enhancing; due to increased status and economic resources at different life stages, and health endangering; due to increased stress and a move towards masculine eating, drinking and smoking habits. For men, increased competition with women may be a stressor, while increased female employment also leads to economic prosperity for society at large as well as for the individual family unit, alleviating the burden of the male breadwinner. This means that the net effect may go in either direction.

An alternative hypothesis is that cluster differences are due to other societal factors such as economic development or income inequality, or health behaviours unrelated to achieved gender equality.

Specific policy indicators were chosen not for their purported health effects, but to measure aspects of Sainsbury’s concept *gender policy regimes,* with gender policy being defined as policy “associated with a certain *gender ideology*, that describe actual or preferred relations between women and men, principles of entitlement, and policy construction” [56]. Therefore it is difficult to make predictions regarding the health impact of specific policies. Also, single policies don’t appear in a vacuum but are part of a policy package, making it difficult to distinguish the influence of a specific policy from the influence of a gender policy regime in general. Nonetheless, we have included specific indicators in order to investigate whether cluster differences found are reflected also in estimates for policy indicators. This also links back to the ongoing discussion whether investigating country clusters or indicators is the best approach.

## Data and methods

### Data

#### Exposures

The country clusters investigated here were developed in an earlier policy study [58]. Data for the period 1973–2008 regarding taxes, parental leave, pensions, social insurances and social services in kind were included and cluster analysis was performed for 1979, 1989, 1999 and 2004. All 22 countries who were members of the OECD at the beginning of the period were included. The main data source was the International Social Security Association’s publication *Social Security Programs Throughout the World* (SSPTW). Additional information was taken from OECD’s Social Expenditure Database, the SCIP (Social Citizenship Indicator Program) database and OECD’s *Taxing wages*. Further information was also retrieved directly from the concerned countries to ensure the validity of data. See Additional file 1, Table 1 for complete presentation of the data.

**Table 1:** Country clusters in 2004: included countries and defining features

	**Male breadwinner**	**Compensatory breadwinner**	**Universal citizen**	**Earner-carer**
Generous parental leaves				**√**
High social services				**√**
Separate taxation		**√**	**√**	**√**
High pension universality			**√**	**√**
High monetary support to breadwinner	**√**	**√**		
Compensatory measures in pension system	(**√**)	**√**		

Male breadwinner: USA, Greece, Portugal, Spain, Switzerland, Japan, Germany, Ireland, France. Compensatory breadwinner: UK, Italy, Austria, Belgium, Canada. Universal citizen: Australia & New Zealand. Earner-carer: Norway, Iceland, Sweden, Denmark, Finland, Netherlands.

Indicators were chosen to reflect aspects of Sainsbury’s concept gender policy regimes. Sainsbury originally proposed two ideal types: the *male breadwinner* and the individual earner-carer regime [56] (from here on abbreviated to *earner-carer*). The male breadwinner regime is characterised by a gender ideology of male privilege based on a gendered division of labour. In the earner-carer regime the preferred relations between women and men are shared roles and obligations, leading to equal rights. Sainsbury later introduced a third model, the *separate gender roles* regime, with social rights attached to the role as either male family provider or female caregiver [59]. The empirical findings support the existence of the first two, but aspects of the separate gender roles regime - compensatory measures in the pension system, and benefits for caring activities - were not present simultaneously [58]. Instead, some countries are classified as compensatory breadwinner countries and towards the end of the period, the two antipodean countries were found in their own cluster (*universal citizen*). In this study, the cluster solution for 2004 is the main predictor. This represents the cluster of destination for the 22 OECD countries. The main reason for the selection of the 2004 cluster solution is that for this year, information on confounders was available for all countries. The included countries and the defining features of the clusters can be found in Table 1.

In short, the male breadwinner countries score higher on monetary support to a sole breadwinner through tax allowances or tax credits, and they also favour a single earner family through treating the household as a single tax unit. They score medium on some compensatory measures in the pension system - credited years for child care - but have low gender differences in retirement age (illustrated by a tick within parentheses). Compensatory breadwinner countries mostly have separate taxation, and they are high performers on compensatory measures in the pension system. The two universal citizen countries are characterised by separate taxation and a universal pension system where no contributions are required. The earner-carer countries are characterised by generous parental leaves, high social services expenditure, separate taxation and high pension universality. The gender policy clusters partly overlap with an expanded welfare state framework (see for example Eikemo et al. [13] or Bambra et al. [11]). This is evident for the earner-carer/social democratic/scandinavian cluster (with the Netherlands as an outlier). The two universal citizen countries correspond to Castles & Mitchells radical welfare states [60], with relatively generous and inclusive means-tested benefits. The male breadwinner cluster, on the other hand, consists of both liberal/anglo-saxon, conservative/bismarckian and Southern European countries and the same goes for the compensatory breadwinner cluster.

### Outcomes

Mortality data comes from WHO Europe’s Health for All database, except for the five non-European countries, where data was collected from WHOs mortality database, and standardised by the authors. The outcomes are mortality from external causes (V00-Y89 in ICD10, B47-B56 in ICD9) and circulatory disease (I00-I99 in ICD10, B25-B30 in ICD9). These are expressed as age standardised (to the European standard population) mortality rates per 100 000. The gender gap was calculated as a standardised measure defined as the absolute difference divided by male mortality * 100. The gender gap for circulatory disease has an overall mean of 63.8 (standard deviation 5.98) and the gender gap for external causes has a mean of 58.8 (standard deviation 7.28).

While external causes of death includes deaths in all age-groups, circulatory disease mortality extends only to those aged 64 years or less. This choice was made based on the fact that external causes is dependent on the behaviour of others as well as that of oneself (e.g. reckless driving may kill infants), and on the fact that all-age circulatory disease mortality is skewed towards the oldest old; we wanted to primarily measure mortality that is avoidable/premature. Due to its small population Iceland has very fluctuating mortality rates and therefore a 3-year moving average was used to smooth the curve for Iceland.

### Confounders and mediators

While economic growth can be seen as necessary but not sufficient for population health [17,61], income inequality is arguably more important for continued health improvements in rich nations [62,63]. Gross Domestic Product (GDP) per capita expressed in USD adjusted for the price level (purchasing power parity) is used an indicator of the economic development level, and the Gini coefficient as an indicator of economic stratification. GDP/capita is available for all years (source: *OECD Factbook* 2009). The Gini coefficient is available since the mid 1970’s but only for eight of the 22 countries. It is updated every 5–10 years and the mid 2000’s is the first time point for which all countries have data. Therefore, the Gini coefficient is included as a time-invariant variable, reflecting the situation around 2005 (source: OECD Social welfare statistics).

Whether cluster differences are mediated through gender equality was analysed by including the GEM, which is an index consisting of the following indicators: female and male shares of parliamentary seats, female and male shares of positions as legislators, senior officials and managers, female and male shares of professional and technical positions (all in relation to the male and female population size), and female and male estimated earned income in relation to a predefined goalpost (PPP US $ 40,000). The first occurrence of the GEM can be found in the 1995 Human development report and it is not updated annually. The GEM was also included as time invariant, reflecting the situation around 2004. For France, there was no data for 2004, and the figure used was the closest in time from 2004.

Some health behaviours that could either be seen as confounders or mediators were also included. These were, for external causes, alcohol consumption based on sales and measured as litre per capita per year, and for circulatory disease, alcohol consumption, total calorie intake per day (also based on sales), and male and female smoking prevalence (based on surveys). Alcohol consumption and calorie intake was unfortunately not divided by sex. Smoking prevalence was not available for all years, with different starting points for different countries (ranging from 1973 to 1997). Missing smoking data (in between end points) was imputed using linear interpolation. All health behaviours come from OECD Health data.

All material for this study consists of data that has been collected from publicly available sources. Due to the aggregate nature of the data, its use does not require ethical approval according to the Stockholm Regional Ethical Review Board (dnr 2011/588-31/5).

## Methods

The data set consists of 22 countries followed across 36 years, 1973–2008, giving a maximum of 792 observations. Since not all confounders were available for all years, there were a number of missing observations (at most 20% for smoking prevalence). Data was analysed using a mixed effects model, a method which can handle unbalanced data sets and that does not require observations nested within higher level units to be unrelated [64,65]. The longitudinal data structure adopted in this article is a particular case of a hierarchical data structure where years are the level 1-units, and countries are the level 2-units [66].

As secular time trends are often strongly (but not necessarily causally) related, time has been included in the model. The modelling approach was to fit time trends using a 3^rd^ degree polynomial, centred at 1990. To avoid over fitting, only the effects of the intercept and of the linear term were allowed to vary between countries. To account for the fact that yearly fluctuations in part depend on the size of a country’s population, average population size (divided by 10^7^) was regarded in the modelling of the intra-country variance (not shown).

The base model contains only time-trends. Model 1 includes also the main predictor the 2004 cluster solution with the male breadwinner cluster as reference group. In model 2, factors related to the economic development level and income distribution (GDP/capita and the Gini coefficient for 2005) have been added as confounders. In model 3 gender equality measured by the GEM for 2004 has been added as a possible mediator, and in model 4 health behaviour indicators have been adjusted for (alcohol, smoking, and calorie intake). The estimates and their respective standard errors are presented in Tables 2 and 3. The estimates for time-varying factors should be interpreted as the (immediate) change in the outcome for each one unit change in the predictor. The estimates for the time-invariant factors reflect the difference in outcome at the intercept (1990) given by a one unit difference in the predictor, and the interaction with time reflect the difference in slope across the whole period for each unit difference in the predictor. Although the gender gap is the focus here, analyses were also performed for male and female absolute levels (see Additional file 1). In these models, the outcome was logged to assume a normal distribution.

The second part of the analyses investigates the relationship between policy indicators and health outcomes. Policy indicators characteristic of those clusters that were significantly different from the reference group male breadwinners were included together with confounders (see Table 4), both individually and in the same model. Some indicators were highly skewed due to a large number of zero scores, and a linear relationship cannot be assumed. Therefore these indicators were dichotomised.

**Table 2:** Gender gap differences in external cause mortality with Cluster 2004 as main predictor

	**Base model**	**Model 1**	**Model 2**	**Model 3**	**Model 4**
**Time trend terms**					
Linear	9.79 (2.00)	9.76 (2.01)	18.86 (2.73)	18.87 (2.75)	16.36 (3.03)
Quadratic	−10.76 (1.08)	−10.72 (1.08)	−7.39 (1.30)	−7.38 (1.31)	−9.55 (1.39)
Cubic	−18.81 (4.15)	−18.67 (4.15)	−14.81 (4.38)	−14.79 (4.38)	−18.19 (4.66)
**Time-varying factors**					
GDP/1000 dollars			−0.32 (0.07)	−0.32 (0.07)	−0.25 (0.07)
Alcohol consumption					−0.21 (0.14)
**Time invariant factors**					
Intercept (constant)	59.75 (1.27)	62.32 (1.70)	56.41 (9.13)	56.27 (14.52)	58.04 (15.51)
Universal citizen		−1.81 (3.66)	−2.25 (3.08)	−2.27 (3.27)	−2.66 (3.41)
Compensatory breadwinner		−2.82 (2.60)	−1.86 (2.21)	−1.87 (2.23)	−2.63 (2.35)
Earner-carer		−6.49 (2.45)	−3.43 (2.70)	−3.44 (2.93)	−4.20 (3.12)
Gini 2005 (0–10)			3.43 (2.75)	3.44 (2.90)	3.19 (3.06)
GEM04 (0–10)				0.01 (1.21)	0.06 (1.26)
**Random part**					
Variance (constant)	35.05 (10.62)	31.99 (9.69)	26.73 (8.11)	26.72 (8.10)	26.21 (7.95)
Covariance (linear/constant)	−23.59 (12.39)	−28.98 (12.46)	−29.82 (11.57)	−29.80 (11.57)	−28.64 (11.81)
Variance (linear)	78.27 (24.17)	78.32 (24.19)	74.59 (23.09)	74.59 (23.09)	83.27 (25.75)

Reference category is the male breadwinner cluster. Regression estimates with standard errors.

Model 1: + Cluster 2004 Model 2: + Economic factors Model 3: + GEM 2004 Model 4: + Alcohol consumption. Time centred at 1990.

**Table 3:** Gender gap differences in circulatory disease mortality (0–64 years) with Cluster 2004 as main predictor

	**Base model**	**Model 1**	**Model 2**	**Model 3**	**Model 4**
**Time trend terms**					
Linear	−1.99 (2.28)	5.35 (2.66)	12.79 (2.87)	55.14 (12.18)	48.42 (11.82)
Quadratic	−10.59 (0.76)	−10.61 (0.76)	−7.88 (0.90)	−7.96 (0.90)	−1.77 (1.05)
Cubic	30.55 (2.95)	30.60 (2.95)	34.13 (3.06)	33.97 (3.06)	25.60 (3.22)
**Time-varying factors**					
GDP/1000 dollars			−0.25 (0.05)	−0.25 (0.05)	−0.26 (0.05)
Female smoking (%)					0.04 (0.04)
Male smoking (%)					−0.10 (0.04)
Alcohol consumption/l					0.05 (0.11)
Calorie intake/100					0.13 (0.10)
**Time invariant factors**					
Intercept (constant)	64.54 (0.98)	62.70 (1.23)	85.03 (8.01)	66.43 (11.89)	73.08 (12.19)
Universal citizen		−1.01 (2.88)	−1.33 (2.72)	−2.96 (2.66)	−3.41 (2.60)
Compensatory breadwinner		1.09 (2.05)	0.64 (1.97)	0.16 (1.84)	−0.47 (1.80)
Earner-carer		6.17 (1.94)	3.11 (2.39)	1.37 (2.41)	1.80 (2.42)
Gini 2005 (0–10)			−5.62 (2.43)	−4.03 (2.37)	−4.45 (2.27)
GEM04 (0–10)				1.88 (0.98)	0.93 (1.01)
**Change over time**					
Universal citizen		−7.18 (6.19)	−8.30 (5.82)	−2.84 (4.90)	−0.05 (4.64)
Compensatory breadwinner		−9.04 (4.40)	−9.00 (4.13)	−6.86 (3.35)	−5.91 (3.18)
Earner-carer		−17.05 (4.16)	−16.42 (3.91)	−7.63 (3.98)	−7.23 (3.85)
GEM04 (0–10)				−6.06 (1.71)	−5.54 (1.64)
**Random part**					
Variance (constant)	20.83 (6.31)	13.48 (4.09)	12.01 (3.65)	10.29 (3.13)	9.58 (2.94)
Covariance (linear/constant)	−18.50 (10.97)	−1.77 (6.19)	−3.46 (5.54)	2.61 (4.09)	5.20 (3.87)
Variance (linear)	109.15 (33.25)	61.07 (18.64)	53.70 (16.53)	33.82 (10.48)	28.69 (9.45)

Reference category is the male breadwinner cluster. Regression estimates with standard errors.

Model 1: + Cluster 2004 Model 2: + Economic factors Model 3: GEM 2004 Model 4: + Smoking, alcohol consumption and calorie intake. Time centred at 1990.

Autocorrelation, i.e. similar departure from an individual country’s estimated trend curve for observations close in time, violates assumptions of independence between residuals and may bias estimates [67]. In order to include an autocorrelation function, data must be balanced (no missing observations) [68], and therefore this was done on a subset of data (1973–2003). For policy indicators with missing observations for more years/countries this was even more limited. Generally, inclusion of the autocorrelation function does not change estimates more than marginally. However, for circulatory disease mortality, child credits estimates decreased by 22%. All analyses were performed using MlWin 2.22 [69].

## Results

Descriptive results regarding trends and cluster differences may be found in Table 5. Significant cluster differences for the gender gap in external cause mortality is found for the earner-carer cluster, which has a gender gap in 1990 6.5 units lower than that in the male breadwinner cluster (Table 2). This finding is based on higher mortality among women, and lower mortality among men in this cluster (Additional file 1). Cluster differences in absolute levels are however small and insignificant. Introducing GDP/capita leads to a large attenuation of cluster differences in the gender gap, and these are no longer significant. Adding the GEM does not change estimates further. For male mortality however, the GEM is associated with a significant decrease over time.

For circulatory disease mortality, results for the gender gap are based on a small and insignificant relative mortality increase among women in earner-carer countries and a relative decrease among men in earner-carer and compensatory breadwinner countries compared to the reference group (Additional file 1). Significant cluster differences for the gender gap can be found for the earner-carer cluster, which has a 6.2 unit higher gender gap in 1990 and a decrease across the whole period which is 17.1 units steeper than that of the male breadwinner cluster (Table 3). The compensatory breadwinner cluster also has a significantly steeper decrease than the reference group (−9.0). The GEM acts as a strong mediator for the earner-carer cluster. One may note that male smoking prevalence is (illogically) negatively related to the gender gap in circulatory disease.

For the second part of our analyses, we investigated earner-carer indicators and for circulatory disease mortality also indicators that distinguish the compensatory breadwinner cluster from the male breadwinner cluster (Table 4). For external causes, the maternity score, reserved paternity leave (dichotomised), social services expenditure and universal basic pensions (dichotomised) are significantly and negatively related to the gender gap. When all earner-carer indicators were included in the same model, reserved paternity leave, social services expenditure and universal basic pensions remain associated with a decreased gender gap. For absolute levels of mortality it was found that after full adjustment social services expenditure and universal basic pensions are associated with an increase of external cause mortality among women (Additional file 1). When all earner-carer indicators were included together, also reserved paternity leave becomes significant. For men social services expenditure is weakly associated with higher mortality while all others are negatively but insignificantly related to the outcome.

**Table 4:** Associations between policy indicators and the gender gap differences in external cause and circulatory disease mortality

**Policy indicators**	**Model 1**	**Model 2**	**Model 3**	**Model 4**	**All earner-carer**	**All compensatory breadwinner**
**External cause mortality**						
Maternity score (weeks*RR)	−0.09 (0.03)	−0.09 (0.03)	−0.09 (0.03)	−0.09 (0.03)	−0.04 (0.04)	
Reserved paternity leave >=2 wks	−2.01 (0.45)	−1.77(0.45)	−1.76 (0.45)	−1.82 (0.46)	−1.58 (0.51)	
Social services (% of GDP)	−0.95 (0.34)	−0.98 (0.35)	−0.98 (0.36)	−0.99 (0.37)	−0.81 (0.36)	
Separate taxation	−1.98 (2.32)	−0.76 (1.89)	−1.07 (1.96)	−1.51 (2.07)	−1.01 (1.96)	
Min pension requirement <=1 yr	−3.80 (1.23)	−3.13 (1.23)	−3.15 (1.28)	−2.97 (1.31)	−6.59 (2.53)	
**Circulatory disease mortality**						
Maternity score (weeks*RR)	0.04 (0.02)	0.02 (0.02)	0.03 (0.02)	0.04 (0.03)	0.05 (0.03)	
Reserved paternity leave >=2 wks	0.23 (0.35)	0.13 (0.35)	0.18 (0.35)	0.27 (0.37)	0.09 (0.40)	
Social services (% of GDP)	0.19 (0.25)	−0.31 (0.27)	−0.22 (0.27)	−0.04 (0.29)	−0.13 (0.29)	
Separate taxation	−0.56 (1.87)	−2.39 (1.58)	−1.44 (1.61)	−1.84 (1.59)	−2.10 (1.55)	
Min pension requirement <=1 yr	1.59 (0.95)	0.97 (0.98)	0.79 (1.00)	1.00 (2.07)	1.37 (1.95)	
Child credits >= 4 yrs/child	−1.11 (0.38)	−1.15 (0.37)	−1.10 (0.37)	−0.81 (0.38)		−0.74 (0.38)
Retiregap>=1 yr	−0.04 (0.53)	0.08 (0.52)	0.08 (0.51)	−0.29 (0.56)		−0.31 (0.60)
Extended leave score >= 10	−0.29 (0.34)	−0.40 (0.33)	−0.39 (0.33)	−0.95 (0.33)		−0.90 (0.33)

Regression estimates with standard error.

For circulatory disease mortality (0–64 years), no earner carer indicators are associated with the gender gap (Table 4). However, reserved paternity leave, social services expenditure and separate taxation are all associated with increased mortality for both men and women. Basic universal pensions are however related to decreased mortality for both men and women, enhanced after adjustment for smoking. For compensatory breadwinner indicators, high pension child credits and a high extended leave score (dichotomised) are associated with a decreased gender gap. For absolute levels, a high extended leave score is related to increased mortality for both men and women, and this association is stronger for women (Additional file 1). High child credits are weakly associated with higher mortality among women, while the retirement age gender gap is associated with lower mortality among men.

## Discussion

For external cause mortality, the earner carer cluster had, as hypothesised, a somewhat lower gender gap than the male breadwinner cluster. This difference was related to several of the policies characteristic of the earner-carer cluster; maternity and paternity leave, social services expenditure and universal basic pensions. The effect on gender gaps was generally due to a positive association with female mortality. Increased exposure to both accidents and violence for women could be a side effect from their increased participation in public life. Also the effect of universal basic pensions could be seen as hinging on increased opportunities for social participation. This is consistent with research showing that some causes of death, e.g. due to traffic accidents, are higher during boom years [70,71]. For specific policies, unlike cluster differences, associations remained also after adjustment for GDP/capita, which means that economic growth is not the only pathway. Here, it would be beneficial to investigate more specific causes of death to determine the mechanisms leading to higher female mortality^a^.

For circulatory disease mortality (0–64 years) both the earner-carer and the compensatory breadwinner clusters experience a larger decrease in male mortality than the male breadwinner cluster, leading to a shrinking gender gap. Additional analyses were performed for circulatory disease mortality in all ages, to see whether results were dependent on the age distribution of these deaths (not shown). The only cluster with a significantly decreasing gender gap in all ages over time compared to the reference group is the universal citizen cluster, due to a larger relative decrease in male mortality. Thus, the decrease in male circulatory mortality achieved in the earner-carer and the compensatory breadwinner clusters is primarily due to a decrease of premature deaths. Earner-carer indicators were however not related to gender gaps in circulatory disease mortality, as most indicators (apart from universal basic pensions which were related to decreased mortality) were associated with higher male as well as female mortality. This is in contrast to the results for cluster differences, and could be interpreted as cluster differences being related to policies not included here. As previously noted, the earner-carer cluster overlaps with the social democratic cluster which is characterised by universality (here represented by universal basic pensions) and benefit generosity (not included). However, income inequality was not a confounder for cluster differences. After adjustment for gender equality measured by the GEM, the decrease in the gender gap which initially was almost twice as large for the earner-carer cluster was similar between the earner-carer and the compensatory breadwinner clusters. A possible pathway could go through shared breadwinning, which could be both stress-reducing for men and lead to higher household income.

**Table 5:** External cause (all ages) and Circulatory disease (0–64 years) mortality (SMR/100 000) and standardised gender gap in 1973–2003 * by gender policy cluster in 2004

**Gender policy cluster**	**Mortality cause**		**1973**	**1981**	**1990**	**1999**	**2003**	**% change**
**Male breadwinner**	External causes	Men	99.06	89.38	79.05	65.46	61.51	−37.9
		Women	41.30	35.94	30.45	24.15	22.92	−44.5
		Stand difference	57.95	59.25	61.23	63.38	62.95	+8.6
	Circulatory	Men	143.09	122.84	94.13	70.78	62.16	−56.6
	disease	Women	65.32	49.36	35.41	26.25	22.56	−65.5
		Stand difference	53.78	59.52	62.70	63.38	64.30	+19.6
**Universal citizen**	External causes	Men	100.97	80.53	75.61	60.21	55.41	−45.1
		Women	50.49	35.18	27.86	24.83	23.38	−53.7
		Stand difference	50.04	56.37	63.05	58.76	57.93	+15.8
	Circulatory	Men	229.41	169.87	107.46	66.15	51.62	−77.5
	disease	Women	94.40	65.58	43.74	26.13	20.26	−78.5
		Stand difference	58.83	61.45	59.80	60.77	61.07	+3.81
**Compensatory**	External causes	Men	102.75	90.24	71.45	60.71	56.31	−45.2
**breadwinner**		Women	45.78	39.49	28.84	24.02	23.07	−49.6
		Stand difference	54.18	55.31	59.57	60.39	59.00	+8.9
	Circulatory	Men	166.74	144.80	97.50	71.74	56.51	−66.1
	disease	Women	67.04	52.97	35.37	27.98	21.18	−68.4
		Stand difference	59.12	63.26	63.76	61.21	62.65	+5.97
**Earner-carer**	External causes	Men	103.38	85.68	81.39	65.65	61.49	−40.5
		Women	45.75	36.03	32.52	26.36	25.19	−44.9
		Stand difference	53.76	56.05	57.54	57.92	57.04	+6.1
	Circulatory	Men	167.99	152.69	108.65	70.50	58.87	−65.0
	disease	Women	54.22	43.02	34.38	24.04	20.25	−62.7
		Stand difference	67.02	71.50	67.51	65.09	64.74	−3.40

* 2003 is the last year for which all countries have data.

### Methodological considerations

The strengths of this study are the inclusion of many countries over an extended period of time, most policy indicators and mortality outcomes are available for each year 1973–2008, and the inclusion of both country clusters and specific policies. Further, there is an inclusion both of economic factors and health behaviours. However, all confounders were not ideally measured. The Gini coefficient and the GEM were included as time invariant, alcohol consumption and calorie intake were not divided by sex, and smoking prevalence was available from the start for only a few countries. Other important exposures that were not included are physical activity and eating habits. However, including fat consumption rather than calorie intake did not affect results (not shown).

One may note that the GEM does not cover important aspects of gender equality such as division of labour within the household, in addition to gender equality in certain (mostly top-level) political and occupational indicators. Perhaps the GEM should be supplemented with indicators measuring women’s status as an absolute concept [72], or gender equality in more basic welfare resources, reflecting what Korpi terms ‘agency poverty’[73]. Indicators of interest could include the female employment rate, percentage women in poverty, voter turnout, and marginal job attachment.

Several factors had to be included as time invariant as time-series data was lacking. These time-invariant variables (the GEM, the Gini coefficient) were set at 2004–05 as all countries had data for this time which means that a country’s value at the end of the period is set as a predictor of differences in 1990 (the centre) and of change over time. Also the cluster solution in this study mirrors only the situation in 2004. It is not immediately obvious that this is the best model, especially if values have changed much over time. However, if the time-invariant factors capture enduring aspects, the existence of time lags should be less of a problem.

Specific indicators of gender regimes were not primarily chosen to act as health predictors nor were all aspects of Sainsbury’s gender policy regime framework possible to include. Employment and wage policies such as antidiscrimination policies and paid components for caring in the home could have been incorporated, had better data been available. Inclusion of more indicators reflecting benefit universality could also have a special bearing on women’s opportunities. Moreover, the quality of data for several policy indicators has increased with time pointing to the possibility of undiscovered errors, especially prior to the 1990s [58].

The existence of lagged effects may concern both policy uptake (cultural lags) and policy lags (societal change comes first) [74]. Time lags are indeed probable between all our predictors and health outcomes. An empirical difficulty relates to the availability of data further back in time and will differ between factors. A theoretical difficulty relates to finding the proper lag, which will depend on the health outcome studied, with external cause mortality being more immediate and circulatory disease mortality requiring decades of exposure. (Immediate policy effects are thus improbable for circulatory disease mortality). When the lag time is expected to be very long incorporating a proper lag structure may be difficult as we lose a fair amount of data. If the exposure is stable over time, specifying a lag time may not be necessary [75], but as we know policies have changed drastically over time. Finally, it is important to note that as individual-level data was not incorporated it has not been able to explore suggested mechanisms or to account for confounding at the individual level.

A different modeling strategy from including time trends is to transform the outcome using simple differencing, so that yearly changes are analysed. With this strategy almost all of the variance was still found between time points. By including time trends however, the intra-class correlation (a measure of the similarity between, in this case, years within countries) was for example 93% for female circulatory disease mortality and 85% for female external cause mortality implying that most of the remaining variance is found between countries. As our interest lies mostly in explaining country/cluster differences including time trends seems like the best option.

## Conclusion

To sum up, the results for external cause mortality are consistent regardless of which approach, cluster or indicator, is used. Results are also in concordance with the hypothesis that women become more exposed to risks of accident and violence when they are economically more active. For circulatory disease mortality, the results differ between the two approaches. There is a decrease in male mortality in the earner-carer and compensatory breadwinner cluster while single policy indicators (except for universal basic pensions which is related to a decrease) are related to increased mortality. As stated earlier, it is possible that cluster differences are explained by unmeasured policies or other societal trends which have not been accounted for. However, much of the male mortality decline in the earner-carer cluster was related to achieved gender equality, while income inequality was not a confounder. We suggest including policy indicators as indices to get a better idea of the impact of the whole policy package. In further studies we would also suggest including general welfare regime indicators alongside gender policy in order to understand these discrepant findings.

## Endnotes

^a^The main dimensions of welfare regimes, as outlined by Esping-Andersen, are social rights in terms of their capacity for decommodification, the redistributive effect of welfare states, and state-market relations in welfare production and distribution, and the three original clusters are the social democratic, the liberal and the corporatist [76]. The welfare regime typology has also been modified to include more countries or additional aspects [28,60,77].

^b^Gender equality of economic participation, political participation and power over economic resources.

^c^The Human Development Index (life expectancy, gross enrolment ratio in education, literacy rate, earned income) adjusted for gender inequality in each dimension.

^d^This could however also lead to problems with reliability between ICD-versions and between countries.

## Competing interests

The authors declare that they have no competing interests.

## Authors’ contributions

MB carried out all data collection and preparation and was the main author. BB and SM were involved in the design and layout of the study, including choice of measures, participated in the interpretation of results and revised the final manuscript. MB and APL performed all data analyses in collaboration. All authors have read and approved the current manuscript.

## Pre-publication history

The pre-publication history for this paper can be accessed here:

http://www.biomedcentral.com/1471-2458/12/969/prepub

## Supplementary Material

Additional file 1 Additional tables. Click here for file
