# Supplementary material for: Is gender policy related to the gender gap in external cause and circulatory disease mortality? A mixed effects model of 22 OECD countries 1973–2008
Source: BMC Public Health. 2012 Nov 12;12:969. doi: 10.1186/1471-2458-12-969 (PMC3560252; doi:10.1186/1471-2458-12-969)
Supplement: Additional file 1 — Additional tables. [file 1471-2458-12-969-S1.docx]

**Appendix 1. Additional tables**

**Table 1.** Policy indicators, confounders/mediators and mortality outcomes.

|  | **Measurement** | **Source** | **Year** | **Comment** |
| --- | --- | --- | --- | --- |
| **Gender policy indicator** | |  |  |  |
| Parental leave | Maternity score (weeks*replacement rate) | SSPTW | 1973-2008 |  |
|  | Reserved and paid paternity leave (weeks) | = | = |  |
| Social services expenditure | Benefits in kind to families and in old age (expenditure as % of GDP) | Social Expenditure Database | 1979-2005 | ICE missing 1980-89 |
| Separate/joint taxation | Individuals taxed separately (yes/no) | Taxing Wages | 1979-2008 | = |
| Monetary support to breadwinner | Marriage subsidy (% of couple take-home pay) | SCIP, Taxing Wages | 1970-2008 | POR & ICE missing all years |
|  | Dependent-wife supplement (0-5) | SSPTW | 1973-2008 |  |
| Pension universality | Minimum pension requirement (years) | = | = |  |
|  | Maximum pension requirement (years) | = | = |  |
| Gendered compensatory measures | Retirement-age gender gap (years) | = | = |  |
| in pension system | Childcare credits (years for two children) | = | = | ITA & POR missing1994-2008 |
| Paid component to caregiver in the home | Extended leave score (weeks*replacement rate) | = | = |  |
| **Confounder/mediator** |  |  |  |  |
| GDP | PPP US $/capita | OECD Factbook | 1973-2008 |  |
| Gini coefficient | 0-10 | OECD Social welfare statistics | 2005 |  |
| GEM | 0-10 | HDR 2005 |  | FRA 2007 |
| Alcohol consumption | Litre/capita | OECD Health Data | 1973-2008 |  |
| Calorie intake | Calories/capita/day | = | = |  |
| Smoking | Male and female prevalence | = |  | 20 % missing data |
| **Mortality outcome** |  |  |  |  |
| External causes (ICD9: B47-B56 | Number of cases / 100.000 | WHO Europe’s | 1973-2008 | Many missing |
| ICD10: V00-Y89) | Gender gap -100 - 100 | Health for All |  | 2004-08 |
| Circulatory disease (ICD9: B25- | Number of cases / 100.000 | database + WHOs |  | = |
| B30, ICD10: I00-I99) | Gender gap -100 - 100 | mortality database |  |  |

**Table 2**. External cause mortality (logged) among women with Cluster 2004 as main predictor.

Reference category is the male breadwinner cluster. Regression estimates with standard errors.

|  | **Base model** | | **Model 1** | | **Model 2** | | **Model 3** | | **Model 4** | |
| --- | --- | --- | --- | --- | --- | --- | --- | --- | --- | --- |
| **Time trend terms** |  |  | |  | |  | |  | |  |
| Linear | -0.82 (0.06) | -0.82 (0.06) | | -1.06 (0.09) | | -1.06 (0.09) | | -0.97 (0.10) | |  |
| Quadratic | 0.21 (0.04) | 0.21 (0.04) | | 0.11 (0.05) | | 0.11 (0.05) | | 0.22 (0.05) | |  |
| Cubic | 0.37 (0.16) | 0.37 (0.16) | | 0.27 (0.17) | | 0.27 (0.17) | | 0.47 (0.18) | |  |
| **Time-varying factors** |  |  | |  | |  | |  | |  |
| GDP/1000 dollars |  |  | | 0.01 (0.00) | | 0.01 (0.00) | | 0.01 (0.00) | |  |
| Alcohol consumption |  |  | |  | |  | | 0.01 (0.01) | |  |
| **Time invariant factors** |  |  | |  | |  | |  | |  |
| Intercept (constant) | 3.37 (0.04) | 3.33 (0.07) | | 4.37 (0.38) | | 3.80 (0.59) | | 3.65 (0.58) | |  |
| Universal citizen |  | 0.03 (0.16) | | 0.05 (0.13) | | 0.00 (0.13) | | 0.01 (0.13) | |  |
| Compensatory breadwinner |  | 0.03 (0.11) | | -0.00 (0.09) | | -0.01 (0.09) | | -0.01 (0.09) | |  |
| Earner-carer |  | 0.09 (0.10) | | -0.17 (0.11) | | -0.24 (0.12) | | -0.16 (0.12) | |  |
| Gini 2005 (0-10) |  |  | | -0.36 (0.12) | | -0.33 (0.12) | | -0.29 (0.12) | |  |
| GEM04 (0-10) |  |  | |  | | 0.06 (0.05) | | 0.06 (0.05) | |  |
| **Random part** |  |  | |  | |  | |  | |  |
| Variance (constant) | 0.04 (0.01) | 0.04 (0.01) | | 0.03 (0.01) | | 0.03 (0.01) | | 0.03 (0.01) | |  |
| Covariance (linear/constant) | 0.01 (0.01) | 0.00 (0.01) | | 0.02 (0.01) | | 0.02 (0.01) | | 0.02 (0.01) | |  |
| Variance (linear) | 0.06 (0.02) | 0.06 (0.02) | | 0.06 (0.02) | | 0.06 (0.02) | | 0.06 (0.02) | |  |

Model 1: + Cluster 2004 Model 2: + Economic factors Model 3: + GEM 2004 Model 4: + Alcohol consumption. Time centred at 1990.

**Table 3**. External cause mortality (logged) among men with Cluster 2004 as main predictor.

Reference category is the male breadwinner cluster. Regression estimates with standard errors.

|  | **Base model** | | **Model 1** | | **Model 2** | | **Model 3** | | **Model 4** | |
| --- | --- | --- | --- | --- | --- | --- | --- | --- | --- | --- |
| **Time trend terms** |  |  | |  | |  | |  | |  |
| Linear | -0.60 (0.05) | -0.60 (0.05) | | -0.70 (0.07) | | 0.03 (0.35) | | 0.14 (0.34) | |  |
| Quadratic | -0.02 (0.03) | -0.02 (0.03) | | -0.08 (0.04) | | -0.09 (0.04) | | -0.04 (0.04) | |  |
| Cubic | -0.06 (0.12) | -0.06 (0.12) | | -0.15 (0.13) | | -0.16 (0.13) | | -0.09 (0.14) | |  |
| **Time-varying factors** |  |  | |  | |  | |  | |  |
| GDP/1000 dollars |  |  | | 0.00 (0.00) | | 0.00 (0.00) | | 0.00 (0.00) | |  |
| Alcohol consumption |  |  | |  | |  | | 0.01 (0.00) | |  |
| **Time invariant factors** |  |  | |  | |  | |  | |  |
| Intercept (constant) | 4.29 (0.05) | 4.32 (0.08) | | 4.54 (0.52) | | 3.97 (0.82) | | 3.80 (0.81) | |  |
| Universal citizen |  | -0.08 (0.18) | | -0.07 (0.18) | | -0.11 (0.18) | | -0.10 (0.18) | |  |
| Compensatory breadwinner |  | -0.02 (0.13) | | -0.09 (0.13) | | -0.10 (0.13) | | -0.09 (0.12) | |  |
| Earner-carer |  | -0.02 (0.12) | | -0.09 (0.15) | | -0.15 (0.17) | | -0.10 (0.16) | |  |
| Gini 2005 (0-10) |  |  | | -0.09 (0.16) | | -0.05 (0.16) | | -0.03 (0.16) | |  |
| GEM04 (0-10) |  |  | |  | | 0.06 (0.07) | | 0.06 (0.07) | |  |
| **Change over time** |  |  | |  | |  | |  | |  |
| GEM04 (0-10) |  |  | |  | | -0.10 (0.05) | | -0.11 (0.04) | |  |
| **Random part** |  |  | |  | |  | |  | |  |
| Variance (constant) | 0.05 (0.02) | 0.05 (0.02) | | 0.05 (0.02) | | 0.05 (0.02) | | 0.05 (0.01) | |  |
| Covariance (linear/constant) | -0.01 (0.01) | -0.01 (0.01) | | -0.01 (0.01) | | -0.00 (0.01) | | -0.00 (0.01) | |  |
| Variance (linear) | 0.05 (0.01) | 0.05 (0.01) | | 0.05 (0.02) | | 0.04 (0.01) | | 0.04 (0.01) | |  |

Model 1: + Cluster 2004 Model 2: + Economic factors Model 3: + GEM 2004 Model 4: + Alcohol consumption. Time centred at 1990.

**Table 4**. Circulatory disease mortality (logged) among women with Cluster 2004 as main predictor.

Reference category is the male breadwinner cluster. Regression estimates with standard errors.

|  | **Base model** | **Model 1** | **Model 2** | **Model 3** | **Model 4** |
| --- | --- | --- | --- | --- | --- |
| **Time trend terms** |  |  |  |  |  |
| Linear | -1.26 (0.07) | -1.21 (0.08) | -1.06 (0.10) | -1.06 (0.10) | -1.01 (0.11) |
| Quadratic | -0.16 (0.03) | -0.16 (0.08) | -0.08 (0.04) | -0.07 (0.04) | -0.05 (0.05) |
| Cubic | -0.51 (0.11) | -0.52 (0.03) | -0.36 (0.12) | -0.36 (0.12) | -0.40 (0.15) |
| **Time-varying factors** |  |  |  |  |  |
| GDP/1000 dollars |  |  | -0.01 (0.00) | -0.01 (0.00) | -0.01 (0.00) |
| Female smoking (%) |  |  |  |  | 0.00 (0.00) |
| Alcohol consumption/l |  |  |  |  | -0.00 (0.01) |
| Calorie intake/100 |  |  |  |  | -0.01 (0.00) |
| **Time invariant factors** |  |  |  |  |  |
| Intercept (constant) | 3.54 (0.05) | 3.53 (0.08) | 2.30 (0.41) | 1.54 (0.63) | 2.04 (0.65) |
| Universal citizen |  | 0.20 (0.18) | 0.21 (0.15) | 0.14 (0.15) | 0.11 (0.15) |
| Compensatory breadwinner |  | 0.07 (0.13) | 0.13 (0.11) | 0.11 (0.10) | 0.10 (0.10) |
| Earner-carer |  | -0.07 (0.12) | 0.20 (0.13) | 0.13 (0.13) | 0.06 (0.14) |
| Gini 2005 (0-10) |  |  | 0.41 (0.13) | 0.47 (0.13) | 0.43 (0.13) |
| GEM04 (0-10) |  |  |  | 0.08 (0.05) | 0.07 (0.05) |
| **Change over time** |  |  |  |  |  |
| Universal citizen |  | -0.61 (0.19) | -0.63 (0.19) | -0.63 (0.19) | -0.63 (0.18) |
| Compensatory breadwinner |  | -0.12 (0.13) | -0.11 (0.13) | -0.11 (0.13) | -0.04 (0.13) |
| Earner-carer |  | 0.13 (0.13) | 0.16 (0.13) | 0.16 (0.13) | 0.24 (0.13) |
| **Random part** |  |  |  |  |  |
| Variance (constant) | 0.06 (0.02) | 0.05 (0.02) | 0.04 (0.01) | 0.03 (0.01) | 0.03 (0.01) |
| Covariance (linear/constant) | -0.01 (0.02) | 0.01 (0.01) | 0.01 (0.01) | 0.01 (0.01) | 0.01 (0.01) |
| Variance (linear) | 0.10 (0.03) | 0.06 (0.02) | 0.06 (0.02) | 0.06 (0.02) | 0.05 (0.02) |

Model 1: + Cluster 2004 Model 2: + Economic factors Model 3: GEM 2004 Model 4: + Smoking, alcohol consumption and calorie intake. Time centred at 1990.

**Table 5**. Circulatory disease mortality (logged) among men with Cluster 2004 as main predictor.

Reference category is the male breadwinner cluster. Regression estimates with standard errors.

|  | **Base model** | | **Model 1** | | **Model 2** | | **Model 3** | | **Model 4** | |
| --- | --- | --- | --- | --- | --- | --- | --- | --- | --- | --- |
| **Time trend terms** |  |  | |  | |  | |  | |  |
| Linear | -1.40 (0.07) | -1.15 (0.08) | | -0.88 (0.08) | | -0.06 (0.39) | | 0.25 (0.39) | |  |
| Quadratic | -0.56 (0.03) | -0.56 (0.03) | | -0.43 (0.03) | | -0.43 (0.03) | | -0.46 (0.04) | |  |
| Cubic | 0.75 (0.10) | 0.75 (0.10) | | 0.99 (0.10) | | 0.99 (0.10) | | 1.26 (0.13) | |  |
| **Time-varying factors** |  |  | |  | |  | |  | |  |
| GDP/1000 dollars |  |  | | -0.01 (0.00) | | -0.01 (0.00) | | -0.02 (0.00) | |  |
| Male smoking |  |  | |  | |  | | 0.00 (0.00) | |  |
| Alcohol consumption |  |  | |  | |  | | 0.01 (0.00) | |  |
| Calorie intake/100 |  |  | |  | |  | | 0.01 (0.00) | |  |
| **Time invariant factors** |  |  | |  | |  | |  | |  |
| Intercept (constant) | 4.60 (0.05) | 4.54 (0.07) | | 3.89 (0.43) | | 2.54 (0.61) | | 2.18 (0.66) | |  |
| Universal citizen |  | 0.16 (0.16) | | 0.17 (0.15) | | 0.05 (0.14) | | 0.09 (0.15) | |  |
| Compensatory breadwinner |  | 0.08 (0.11) | | 0.13 (0.11) | | 0.10 (0.10) | | 0.12 (0.10) | |  |
| Earner-carer |  | 0.11 (0.11) | | 0.29 (0.13) | | 0.18 (0.13) | | 0.22 (0.13) | |  |
| Gini 2005 (0-10) |  |  | | 0.25 (0.13) | | 0.38 (0.12) | | 0.37 (0.13) | |  |
| GEM04 (0-10) |  |  | |  | | 0.13 (0.05) | | 0.15 (0.05) | |  |
| **Change over time** |  |  | |  | |  | |  | |  |
| Universal citizen |  | -0.79 (0.18) | | -0.83 (0.16) | | -0.72 (0.16) | | -0.72 (0.15) | |  |
| Compensatory breadwinner |  | -0.35 (0.13 ) | | -0.34 (0.12) | | -0.30 (0.11) | | -0.31 (0.11) | |  |
| Earner-carer |  | -0.32 (0.12) | | -0.28 (0.11) | | -0.11 (0.13) | | -0.12 (0.13) | |  |
| GEM04 (0-10) |  |  | |  | | -0.12 (0.05) | | -0.13 (0.06) | |  |
| **Random part** |  |  | |  | |  | |  | |  |
| Variance (constant) | 0.04 (0.01) | 0.04 (0.01) | | 0.04 (0.01) | | 0.03 (0.01) | | 0.03 (0.01) | |  |
| Covariance (linear/constant) | -0.02 (0.02) | -0.00 (0.01) | | -0.00 (0.01) | | 0.01 (0.02) | | -0.01 (0.01) | |  |
| Variance (linear) | 0.11 (0.03) | 0.05 (0.02) | | 0.04 (0.01) | | 0.03 (0.01) | | 0.03 (0.01) | |  |

Model 1: + Cluster 2004 Model 2: + Economic factors Model 3: GEM 2004 Model 4: + Smoking, alcohol consumption and calorie intake. Time centred at 1990.

**Table 6.** Associations between policy indicators and external cause and circulatory disease mortality among women. Regression estimates with standard errors.

| **Policy indicators** | | **Model 1** | | **Model 2** | | **Model 3** | | **Model 4** | **All earner-carer** | **All compensatory breadwinner** |
| --- | --- | --- | --- | --- | --- | --- | --- | --- | --- | --- |
| **External cause mortality** | |  | |  | |  | |  |  |  |
| Maternity score (weeks*RR) | -0.00 (0.00) | | -0.00 (0.00) | | -0.00 (0.00) | | -0.00 (0.00) | | -0.00 (0.00) |  |
| Reserved paternity leave >=2 wks | 0.03 (0.02) | | 0.02 (0.02) | | 0.02 (0.02) | | 0.02 (0.02) | | 0.04 (0.02) |  |
| Social services (% of GDP) | 0.04 (0.01) | | 0.05 (0.01) | | 0.05 (0.01) | | 0.05 (0.01) | | 0.06 (0.01) |  |
| Separate taxation | -0.00 (0.09) | | -0.07 (0.08) | | -0.06 (0.08) | | -0.02 (0.08) | | -0.09 (0.08) |  |
| Min pension requirement <=1 yr | 0.15 (0.05) | | 0.12 (0.05) | | 0.12 (0.05) | | 0.12 (0.05) | | 0.22 (0.10) |  |
| **Circulatory disease mortality** | | |  | |  | |  | |  |  |
| Maternity score (weeks*RR) | 0.00 (0.00) | | 0.00 (0.00) | | 0.00 (0.00) | | 0.00 (0.00) | | -0.00 (0.00) |  |
| Reserved paternity leave >=2 wks | 0.02 (0.01) | | 0.02 (0.01) | | 0.02 (0.01) | | 0.01 (0.01) | | 0.03 (0.01) |  |
| Social services (% of GDP) | 0.04 (0.01) | | 0.04 (0.01) | | 0.04 (0.01) | | 0.03 (0.01) | | 0.03 (0.01) |  |
| Separate taxation | 0.03 (0.10) | | 0.13 (0.09) | | 0.21 (0.08) | | 0.19 (0.08) | | 0.27 (0.07) |  |
| Min pension requirement <=1 yr | -0.08 (0.04) | | -0.06 (0.04) | | -0.08 (0.04) | | -0.22 (0.11) | | -0.30 (0.09) |  |
| Child credits >= 4 yrs/child | 0.01 (0.01) | | 0.01 (0.01) | | 0.01 (0.01) | | 0.02 (0.01) | |  | 0.01 (0.01) |
| Retiregap>=1 yr | -0.01 (0.02) | | -0.01 (0.02) | | -0.01 (0.02) | | -0.01 (0.02) | |  | -0.01 (0.02) |
| Extended leave score >= 10 | 0.07 (0.01) | | 0.08 (0.01) | | 0.08 (0.01) | | 0.07 (0.01) | |  | 0.07 (0.01) |

**Table 7.** Associations between policy indicators and external cause and circulatory disease mortality among men. Regression estimates with standard errors.

| **Policy indicators** | | **Model 1** | | **Model 2** | | **Model 3** | | **Model 4** | **All earner-carer** | **All compensatory breadwinner** |
| --- | --- | --- | --- | --- | --- | --- | --- | --- | --- | --- |
| **External cause mortality** | |  | |  | |  | |  |  |  |
| Maternity score (weeks*RR) | -0.00 (0.00) | | -0.00 (0.00) | | -0.00 (0.00) | | -0.00 (0.00) | | -0.00 (0.00) |  |
| Reserved paternity leave >=2 wks | -0.02 (0.01) | | -0.02 (0.01) | | -0.02 (0.01) | | -0.02 (0.01) | | -0.00 (0.01) |  |
| Social services (% of GDP) | 0.02 (0.01) | | 0.03 (0.01) | | 0.03 (0.01) | | 0.04 (0.01) | | 0.04 (0.01) |  |
| Separate taxation | -0.08 (0.10) | | -0.10 (0.10) | | -0.10 (0.11) | | -0.07 (0.11) | | -0.12 (0.11) |  |
| Min pension requirement <=1 yr | 0.07 (0.04) | | 0.07 (0.04) | | 0.07 (0.04) | | 0.07 (0.04) | | 0.14 (0.15) |  |
| **Circulatory disease mortality** | | |  | |  | |  | |  |  |
| Maternity score (weeks*RR) | 0.00 (0.00) | | 0.00 (0.00) | | 0.00 (0.00) | | 0.00 (0.00) | | -0.00 (0.00) |  |
| Reserved paternity leave >=2 wks | 0.03 (0.01) | | 0.03 (0.01) | | 0.03 (0.01) | | 0.03 (0.01) | | 0.03 (0.01) |  |
| Social services (% of GDP) | 0.04 (0.01) | | 0.04 (0.01) | | 0.04 (0.01) | | 0.04 (0.01) | | 0.03 (0.01) |  |
| Separate taxation | 0.02 (0.09) | | 0.03 (0.09) | | 0.21 (0.08) | | 0.21 (0.08) | | 0.21 (0.08) |  |
| Min pension requirement <=1 yr | -0.08 (0.03) | | -0.08 (0.03) | | -0.08 (0.03) | | -0.25 (0.11) | | -0.28 (0.10) |  |
| Child credits >= 4 yrs/child | -0.01 (0.01) | | -0.02 (0.01) | | -0.02 (0.01) | | 0.00 (0.01) | |  | -0.00 (0.01) |
| Retiregap>=1 yr | -0.03 (0.02) | | -0.03 (0.02) | | -0.03 (0.02) | | -0.04 (0.02) | |  | -0.05 (0.02) |
| Extended leave score >= 10 | 0.04 (0.01) | | 0.05 (0.01) | | 0.05 (0.01) | | 0.04 (0.01) | |  | 0.04 (0.01) |
